# Association between changes in economic activity and catastrophic health expenditure: findings from the Korea Health Panel Survey, 2014–2016

**DOI:** 10.1186/s12962-020-00233-9

**Published:** 2020-09-16

**Authors:** Hyeon Ji Lee, Doo Woong Lee, Dong-Woo Choi, Sarah Soyeon Oh, Junhyun Kwon, Eun-Cheol Park

**Affiliations:** 1 Department of Public Health, Graduate School, Yonsei University, Seoul, Republic of Korea; 2 Institute of Health Services Research, Yonsei University, 50 Yonsei-ro, Seodaemun-gu, Seoul, 03722 Republic of Korea; 3 Department of Obstetrics & Gynecology, Ewha Womans University, Seoul, Republic of Korea; 4 Department of Preventive Medicine, Yonsei University College of Medicine, 50 Yonsei-ro, Seodaemun-gu, Seoul, 03722 Republic of Korea

**Keywords:** Catastrophic payment, Health expenditures, Economic status, Employment, South Korea

## Abstract

**Background:**

The rate of catastrophic health expenditure (CHE) continues to rise in South Korea. This study examined the association between changes in economic activity and CHE experiences in South Korea.

**Methods:**

This study analyzed the Korea Health Panel Survey data using a logistic regression analysis to study the association between changes in economic activity in 2014–2015 and the participants’ CHE experiences in 2015. The study included a total of 12,454 individuals over the age of 19. The subgroup analyses were organized by sex, age, health-related variables, and household level variables, and the reasons for leaving economic activity.

**Results:**

Those who quit economic activities were more likely to experience CHE than those who continued to engage in economic activities (OR [odds ratio] = 2.10; 95% CI [confidence interval]: 1.31–3.36). The subgroup analysis results, according to health-related variables, showed that there is a tendency to a higher Charlson comorbidity index, a higher OR, and, in groups that quit their economic activities, people with disabilities were more likely to experience CHE than people without disabilities (OR = 5.63; 95% CI 1.71–18.59, OR = 1.82; 95% CI 1.08–3.08, respectively). Another subgroup analysis found that if the reason for not participating in economic activity was a health-related issue, the participant was more likely to experience CHE (active → inactive: OR = 2.40; 95% CI 0.61–9.43, inactive → inactive OR = 1.65; 95% CI 1.01–2.68).

**Conclusions:**

Those individuals who became unemployed were more likely to experience CHE, especially if health problems precipitated the job loss. Therefore, efforts are needed to expand coverage for those people who suffer from high medical expenses.

## Background

Healthcare costs are always incurred according to economic principles. Therefore, patients may have to pay healthcare expenses in accordance with the national healthcare reimbursement systems and can sometimes suffer from financial stress in addition to their illness, and may experience poverty due to having to pay their medical expenses. The existence of patients substantially suffering from medical costs shows the insufficient health coverage of a country.

In 2018, the Organisation for Economic Co-operation and Development’s (OECD) average of the proportion of total healthcare spending financed by the public sector was 73.8%, compared to 59.8% in South Korea, which is second-lowest among the OECD countries (the maximum is 85.5% in Norway, and the minimum 51.5% in Mexico) [1]. In the United States, the proportion of total healthcare spending financed by the public sector is the second-highest among the OECD countries at 14.3% [1], but medical bankruptcy is also a significant problem [2, 3]. South Korea is also not immune to the risk of medical bankruptcy. South Korea is a national health insurance system; however, underinsurance problems continue [4–6]. In addition, South Korea risks increasing the number of people experiencing healthcare expense related distress due to the increasing burden of out-of-pocket payments [1], and the rate of catastrophic health expenditure (CHE) experience continues to rise [7].

Distress from medical costs needs to be considered on a narrower scale as well as on a national level. CHE is different from high-cost medical costs and closely corresponds to the economic capacity of households and individuals [8]. Even when individuals incur high medical costs, those with a sufficient capacity to buffer them can defend themselves against economic difficulties. However, individuals who cannot afford to pay will experience difficulties caused by healthcare expenses. In other words, CHE is a concept that considers the varying healthcare expense burdens between individuals of different economic capacities. To define CHE, economic capacity can be defined in various ways, such as total income, total expenditure, and the poverty line. The share of healthcare expenses has also been presented in a variety of ways, from 5 to 40% [9–12].

Numerous previous studies have explored CHE’s causes to prevent their occurrence. According to the results of these studies, demographic, health-related, and socioeconomic factors, such as region of residence, income level, and economic activity, were identified as factors correlated with catastrophic health expenses [13–15]. Some studies also considered the variable factors that led to CHE occurrence [16, 17]. These studies looked at the relationship between a change in economic activity and CHE, and reported that economic inactivity was linked to CHE. However, these studies were conducted on select population groups, such as people with disabilities, people who have chronic diseases, or heads of households. Therefore, this study’s purpose was to examine the association between changes in economic activity and CHE experience for the general population.

## Methods

### Data collection and study participants

This study analyzed data from the 9th to 11th wave (2014–2016) of the Korea Health Panel Survey (KHPS), which has been conducted annually since 2008 [18] by the Korea Institute for Health and Social Affairs and the National Health Insurance Corporation. The surveyor visits the target household and conducts the interview using Computer Assisted Personal Interviewing methods. They surveyed monthly household consumption expenditures and annual household income based on the previous year according to household ledgers, receipts, or self-reports. Additionally, annual household income was converted to an equalized household income value considering the number of household members. Information on healthcare spending was collected retroactively on a yearly basis using household ledgers, receipts, and insurance usage information to prevent omissions or errors in recall due to the length of time between healthcare service use and the date of the investigation.

In addition, the state of economic activity is surveyed on the last day of the year. The dining out expenses needed to accurately calculate the CHE have been investigated since 2014, and the most recent year when the information was released is 2016. Therefore, the 2014–2016 KHPS data was used to analyze the experience of CHE in 2015 in accordance with changed economic activities from 2014–2015.

Of the 18,130 participants in 2015, 14,530 remained, with those under 19 years removed. Of these, one person was removed from the study population as the CHE could not be calculated, and other participants without information on the control variables were removed, bringing the total number to 13,709. Finally, a total of 12,454 participants were analyzed as after eliminating people whose economic activity was unknown in 2014 or 2015.

### Catastrophic health expenditure experience

The dependent variable is the experience of CHE in 2015. The World Health Organization proposed methods for calculating CHE and defines CHE as a situation when medical expenses are more than 40% of one’s capacity to pay [10]. The method considered a household’s capacity to pay by subtracting essential food expenditures or household subsistence spending from the sum of household spending, and households were defined as experiencing CHE when household out-of-pocket payment on medical expenses accounted for over 40% of the household capacity. If healthcare expenses accounted for more than 40% of the amount that could be spent, then the household was classified as the Yes group who experienced CHE. If household expenses were less than the criteria, then the household was classified as the No group that did not experience CHE. The items on household consumption expenditure in the survey questioned the costs spent over the past year, so the 2016 survey used 2015 expenditure values. Healthcare expenses were defined as expenses for hospitalizations, outpatient services, emergency services, and prescription drugs throughout 2015.

### Changes in economic activity

The independent variable is the change in economic activity in 2014–2015. The status of economic activity was classified as yes if they worked for income purpose and; or no if they did not. As survey was conducted at the end of last year, asking whether the individual participated in economic activity or not. The economic activity on December 31, 2014, surveyed in the 2015 survey, and the economic activity on December 31, 2015, surveyed in the 2016 survey, were categorized into the following four categories: active no change, inactive then active, active then inactive, or inactive no change.

### Control variables

The control variables included individual and household level variables. The variables at the individual level are sex, age, marital status, education level, medical aid, disability status, Charlson comorbidity index (CCI), unmet medical need, current smoking, and alcohol consumption statuses. The CCI was calculated according to the method defined in a previous study and sorted into three categories: 0, 1, 2 + [19]. The variables at the household level are the region of residence, head of household, household income level, lagged household income level (i.e., household income level in 2014), and lagged dependent variable (i.e., CHE experience in 2014). KHPS was surveyed by defining the person who represents the household as a householder, regardless of whether the residence was owned or their income. Based on the survey responses, the head of household was categorized into householder, otherwise non-householder. The household income level and lagged household income level were categorized using equalized income.

### Supplementary analysis

A subgroup analysis of the association between dependent and independent variables was performed according to sex, age, health-related variables (disability status, CCI, unmet medical needs), and household level variables (region, head of household, household income level, lagged household income level, lagged dependent variable) among the covariates. Additionally, to analyze the association between the discontinuation of economic activity by subgroup analysis and the CHE, the interview questionnaire includes the question “Why are you economically inactive?”. The answers for reasons for not engaging in economic activities were categorized as follows and considered as economically inactive factors: family-related factors (housekeeping, upbringing, and care), health-related factors (inability to work, illness or health deterioration, and accidental damage), retirement, other (academic work, job preparation, military service, etc.), and voluntary selection (no will to work or no job search). A sensitivity analysis was also performed by changing the CHE criteria to not only 40%, but also 30%, 20%, and 10% to confirm the results [8, 12].

### Statistical analysis

Using the Chi square test, this study examined the differences between groups according to the independent variables. A *p* value of < 0.05 was considered significant. A logistic regression analysis was performed to investigate the association between changes in economic activity and CHE with calculations expressed as odds ratio (OR) and 95% CI (confidence interval). We checked for multicollinearity in the statistical model through tolerance, variance inflation factors (VIFs), and collinearity diagnostics. In this model, the tolerance values were all under the 0.1 and VIFs were all less than 2.5, and the results of collinearity diagnostics were acceptable; therefore, the results indicate no problem with the correlations between the dependent and independent variables [20]. All statistical analyses were performed using SAS statistical software package version 9.4. (SAS Institute Inc., Cary, NC, USA).

## Results

Table 1 shows the general characteristics of the study population. Of the 12,454 participants, 297 (2.4%) experienced CHE, while 12,157 (97.6%) did not experience CHE in 2015. In terms of the change in economic activity, the percentage of participants with CHE was in the order of active then inactive (4.3%), inactive no change (3.8%), inactive then active (3.3%), and active no change (1.3%), and the result was statistically significant (p = <0.0001).

**Table 1:** General characteristics of the study population

Variables	Total	CHE experience in 2015^a^		P-value
	N (%)	Yes (%)	No (%)	
Economic activity (2014 → 2015)				<.0001
Active → active	6837 (54.9)	87 (1.3)	6750 (98.7)	
Inactive → active	796 (6.4)	26 (3.3)	770 (96.7)	
Active → inactive	611 (4.9)	26 (4.3)	585 (95.7)	
Inactive → inactive	4210 (33.8)	158 (3.8)	4052 (96.2)	
Sex				0.1211
Men	5667 (45.5)	122 (2.2)	5545 (97.8)	
Women	6787 (54.5)	175 (2.6)	6612 (97.4)	
Age				<.0001
19–34	1808 (14.5)	7 (0.4)	1801 (99.6)	
35–49	3619 (29.1)	12 (0.3)	3607 (99.7)	
50–64	3449 (27.7)	56 (1.6)	3393 (98.4)	
65–	3578 (28.7)	222 (6.2)	3356 (93.8)	
Marital status				0.3432
Married	8834 (70.9)	218 (2.5)	8616 (97.5)	
Single, widowed, divorced, separated	3620 (29.1)	79 (2.2)	3541 (97.8)	
Educational level				<.0001
Middle school and below	4126 (33.1)	218 (5.3)	3908 (94.7)	
High school	4473 (35.9)	60 (1.3)	4413 (98.7)	
College and over	3855 (31.0)	19 (0.5)	3836 (99.5)	
Medical aid				0.5929
No	12,051 (96.8)	289 (2.4)	11,762 (97.6)	
Yes	403 (3.2)	8 (2.0)	395 (98.0)	
Disability status				<.0001
No	11,571 (92.9)	253 (2.2)	11,318 (97.8)	
Yes	883 (7.1)	44 (5.0)	839 (95.0)	
Charlson comorbidity index				<.0001
0	10,321 (82.9)	173 (1.7)	10,148 (98.3)	
1	1475 (11.8)	73 (4.9)	1402 (95.1)	
2+	658 (5.3)	51 (7.8)	607 (92.2)	
Unmet medical needs				0.0271
No	9401 (75.5)	208 (2.2)	9193 (97.8)	
Yes	3053 (24.5)	89 (2.9)	2964 (97.1)	
Current smoker				<.0001
No	10,207 (82.0)	270 (2.6)	9937 (97.4)	
Yes	2247 (18.0)	27 (1.2)	2220 (98.8)	
Alcohol consumption				<.0001
No	8176 (65.6)	128 (1.6)	8048 (98.4)	
Yes	4278 (34.4)	169 (4.0)	4109 (96.0)	
Region				<.0001
Urban area	5298 (42.5)	74 (1.4)	5224 (98.6)	
Rural area	7156 (57.5)	223 (3.1)	6933 (96.9)	
Head of household				<.0001
Householder	5986 (48.1)	177 (3.0)	5809 (97.0)	
Non-householder	6468 (51.9)	120 (1.9)	6348 (98.1)	
Household income level				0.0020
Low	5136 (41.2)	151 (2.9)	4985 (97.1)	
Middle	3762 (30.2)	81 (2.2)	3681 (97.8)	
High	3556 (28.6)	65 (1.8)	3491 (98.2)	
Lagged household income level (in 2014)				<.0001
Low	4528 (36.4)	227 (5.0)	4301 (95.0)	
Middle	3952 (31.7)	54 (1.4)	3898 (98.6)	
High	3974 (31.9)	16 (0.4)	3958 (99.6)	
Lagged dependent variable (in 2014)				<.0001
With CHE	292 (2.3)	60 (20.5)	232 (79.5)	
Without CHE	12,162 (97.7)	237 (1.9)	11,925 (98.1)	
Total	12,454 (100.0)	297 (2.4)	12,157 (97.6)	

The p-value is the Chi square test result

^a^*CHE* Catastrophic health expenditure

Table 2 shows the association between economic activity changes and CHE experience. In terms of economic activity, the active → inactive group experienced economic activity that had the highest OR for CHE experience compared to the active → active group (reference group), which was statistically significant (OR = 2.10; 95% CI 1.31–3.36). The inactive → active group and the inactive → inactive group had higher ORs than those of the reference group, and they were statistically significant (OR = 1.83; 95% CI 1.14–2.94, OR = 1.45; 95% CI 1.07–1.97, respectively). In addition, if CHE was experienced in 2014, the OR of experience CHE in 2015 was significantly higher (OR = 4.58; 95% CI 3.27–6.42). A sensitivity analysis of the association between economic activity changes and CHE experience according to differences in the proportion of the medical costs that define CHE showed that even when the criteria were different, the active → inactive group had the highest OR for CHE experience compared to the reference group, and all results were statistically significant. When 10% and 20% were used as the criteria, the OR of the inactive → inactive group was higher than that of the inactive → active group, but when the criteria were 30% and 40%, the opposite trend was shown (Additional file 1: Figure S1).

**Table 2:** Factors associated with CHE

Variables	CHE experience in 2015^a^	
	Adjusted OR	95% CI
Economic activity (2014 → 2015)		
Active → active	1.00	–
Inactive → active	1.83	(1.14–2.94)
Active → inactive	2.10	(1.31–3.36)
Inactive → inactive	1.45	(1.07–1.97)
Sex		
Men	1.00	–
Women	1.00	(0.67–1.52)
Age		
19–34	1.00	–
35–49	0.72	(0.27–1.92)
50–64	2.14	(0.88–5.19)
65–	3.89	(1.60–9.47)
Marital status		
Married	1.00	–
Single, widowed, divorced, separated	0.75	(0.52–1.08)
Educational level		
Middle school and below	2.02	(1.17–3.47)
High school	1.55	(0.91–2.65)
College and over	1.00	–
Medical aid		
No	1.00	–
Yes	0.38	(0.19–0.80)
Disability status		
No	1.00	–
Yes	1.14	(0.80–1.62)
Charlson comorbidity index		
0	1.00	–
1	1.48	(1.10–1.99)
2+	1.82	(1.27–2.59)
Unmet medical needs		
No	1.00	–
Yes	0.85	(0.65–1.11)
Current smoker		
No	1.00	–
Yes	0.72	(0.46–1.12)
Alcohol consumption		
No	1.00	–
Yes	1.05	(0.81–1.37)
Region		
Urban area	1.00	–
Rural area	1.78	(1.35–2.34)
Head of household		
Householder	1.16	(0.78–1.73)
Non-householder	1.00	–
Household income level		
Low	0.95	(0.69–1.29)
Middle	1.03	(0.73–1.45)
High	1.00	–
Lagged household income level (in 2014)		
Low	4.18	(2.42–7.22)
Middle	2.53	(1.43–4.47)
High	1.00	–
Lagged dependent variable (in 2014)		
With CHE	4.58	(3.27–6.42)
Without CHE	1.00	–

All control variables were adjusted

^a^*CHE* Catastrophic health expenditure

Table 3 reports the association between the cause of economic inactivity and the CHE experience, and showed that the ORs of other groups were lower than those of the voluntary selection group (reference group), but in health-related factors, the OR is higher in both active → inactive and inactive → inactive groups. However, the results were statistically significant only in inactive → inactive groups (OR = 2.40; 95% CI 0.61–9.43, OR = 1.65; 95% CI 1.01–2.68, respectively).

**Table 3:** Association between the cause of economic inactivity and the CHE experience in 2015^a^

Variables	Economic activity 2014 → 2015			
	Active → inactive		Inactive → inactive	
	Adjusted OR	95% CI	Adjusted OR	95% CI
Economically inactive factors				
Family related factors	0.20	(0.02–2.56)	0.57	(0.29–1.11)
Health related factors	2.40	(0.61–9.43)	1.65	(1.01–2.68)
Retirement	0.47	(0.08–2.75)	0.79	(0.46–1.35)
Other	0.58	(0.10–3.26)	0.40	(0.13–1.22)
Voluntary selection^b^	1.00	–	1.00	–

All control variables were adjusted

^a^*CHE* Catastrophic health expenditure

^b^Not in education, employment, or training as a voluntary choice

Table 4 shows the results of the subgroup analysis according to the sex, age, health-related, and household-level variables. According to the subgroup analysis by sex, men have the highest OR in the active → inactive group and women in the inactive → active group (OR = 2.59; 95% CI 1.27–5.27, OR = 1.84; 95% CI 1.01–3.33, respectively). In the age subgroup analysis, the OR of the active → inactive group is the highest in the group aged 65 or older (OR = 1.91; 95% CI 1.10–3.31).

**Table 4:** Subgroup analysis of the CHE experience in 2015^a^

Variables	Economic activity 2014 → 2015						
	Active → active	Inactive → active		Active → inactive		Inactive → inactive	
	Adjusted OR	Adjusted OR	95% CI	Adjusted OR	95% CI	Adjusted OR	95% CI
Sex							
Men	1.00	1.58	(0.70–3.57)	2.59	(1.27–5.27)	1.61	(1.00–2.58)
Women	1.00	1.84	(1.01–3.33)	1.68	(0.89–3.18)	1.30	(0.87–1.95)
Age							
19–34	1.00	1.83	(0.13–26.20)	<0.001	<0.001–>999.999	4.77	(0.51–44.84)
35–49	1.00	1.16	(0.11–12.07)	5.01	(0.75–33.39)	0.64	(0.10–3.96)
50–64	1.00	1.83	(0.70–4.81)	1.70	(0.56–5.12)	1.79	(0.91–3.54)
65–	1.00	1.54	(0.85–2.79)	1.91	(1.10–3.31)	1.32	(0.93–1.88)
Disability status							
No	1.00	1.77	(1.08–2.92)	1.82	(1.08–3.08)	1.46	(1.06–2.03)
Yes	1.00	2.24	(0.42–11.93)	5.63	(1.71–18.59)	1.46	(0.62–3.43)
Charlson comorbidity index							
0	1.00	1.46	(0.80–2.69)	1.63	(0.85–3.13)	1.43	(0.98–2.09)
1	1.00	2.04	(0.70–5.98)	1.85	(0.71–4.82)	1.22	(0.66–2.27)
2+	1.00	3.90	(1.13–13.50)	7.47	(2.23–25.07)	2.41	(0.93–6.29)
Unmet medical needs							
No	1.00	1.96	(1.15–3.33)	1.78	(0.96–3.30)	1.39	(0.97–1.99)
Yes	1.00	1.29	(0.42–3.94)	2.65	(1.23–5.71)	1.57	(0.88–2.80)
Region							
Urban area	1.00	2.19	(0.74–6.44)	5.92	(2.51–13.95)	2.14	(1.06–4.30)
Rural area	1.00	1.79	(1.05–3.05)	1.42	(0.78–2.58)	1.37	(0.96–1.93)
Head of household							
Householder	1.00	1.92	(0.99–3.75)	2.73	(1.50–4.94)	1.76	(1.17–2.66)
Non-householder	1.00	1.50	(0.77–2.95)	1.27	(0.57–2.84)	1.06	(0.67–1.67)
Household income level							
Low	1.00	1.96	(1.05–3.66)	2.31	(1.25–4.27)	1.18	(0.77–1.80)
Middle	1.00	3.86	(1.64–9.09)	4.07	(1.63–10.13)	2.89	(1.49–5.60)
High	1.00	<0.001	<0.001–>999.999	0.60	(0.13–2.73)	1.22	(0.64–2.34)
Lagged household income level (in 2014)							
Low	1.00	1.59	(0.91–2.78)	1.85	(1.06–3.22)	1.40	(0.99–1.98)
Middle	1.00	2.50	(0.92–6.76)	3.27	(1.21–8.78)	1.27	(0.59–2.72)
High	1.00	1.41	(0.13–15.37)	2.36	(0.26–21.36)	2.86	(0.77–10.60)
Lagged dependent variable (in 2014)							
With CHE	1.00	1.38	(0.45–4.25)	3.28	(0.57–19.03)	1.25	(0.55–2.82)
Without CHE	1.00	1.80	(1.05–3.08)	2.02	(1.22–3.32)	1.47	(1.05–2.05)

All control variables were adjusted

^a^*CHE* Catastrophic health expenditure

Concerning the subgroup by disability status, the OR of the active → inactive group is the highest for people both with and without disabilities (OR = 5.63; 95% CI 1.71–18.59, OR = 1.82; 95% CI 1.08–3.08, respectively) with the trends for the former being higher than for the latter. There is a tendency that the higher the CCI, the higher the OR. In unmet medical needs, the No group has the highest OR of the inactive → active group, while the Yes group has the highest OR of the active → inactive group (OR = 1.96; 95% CI 1.15–3.33, OR = 2.65; 95% CI 1.23–5.71, respectively).

In terms of household level, the householder group had a higher OR than the non-householder group. The active → inactive group in the householder group has the highest OR (OR = 2.73; 95% CI 1.50–4.94) and is statistically significant; however, in the non-householder group, the inactive → active group shows the highest OR and is not, statistically not significant. According to the household income level, the middle OR group tended to be higher than the low group, and the active → inactive group in both low and middle groups has the highest OR (OR = 2.31; 95% CI 1.25–4.27, OR = 4.07; 95% CI 1.63–10.13, respectively). In lagged household income level (in 2014), the active → inactive group in the low and middle group has the highest OR and the inactive → inactive group in the high group has the highest OR. According to the lagged dependent variable, that is, CHE experience in 2014, the OR of the active-inactive group is the highest in the groups with and without CHE.

## Discussion

This study examined the association between economic activity changes and the experience of CHE. According to the results, when the criteria for CHE was set at 40%, those who discontinue economic activities were more likely to experience CHE than those who continue economic activities. Sensitivity analyses performed by different criteria for defining CHE also showed that the risk that an individual would experience CHE when they quit economic activities tended to be the highest compared to other groups. The expansion of previous studies’ results into general population groups provides further evidence that employment status is linked to CHE. These findings are in line with those of previous studies, as one of the factors precipitating CHE is the cessation of economic activities [15–17]. The results here show that householders are more likely to experience CHE if they lose their jobs.

In addition, according to the sensitivity analysis results, even if the proportion of medical expenses changes in respect to the total household expenses, it is likely that the household will experience CHE if its members cease economic activities. This can be interpreted as unemployment being closely related to medical expenses burden and increasing the risk of experiencing CHE. That is, the size of the denominator is reduced in the formula for calculating CHE. Regardless of the income size resulting from economic activity, unemployment leads to the loss of disposable income, which is likely to lead to CHE.

A further cause to explore is that if the amount of money spent on health care costs increases, there is an additional reason to experience CHE. In other words, the size of the numerator increases in the formula for evaluating CHE. The subgroup analysis of variables related to health status showed that the association between economic activity and CHE experience tended to be stronger in people with a disability, with unmet medical needs, or with a higher CCI (i.e., people with a health-related problem) than relatively healthy people (non-disabled, without unmet medical needs, or 0 of CCI group). In addition, this study showed that individuals were more likely to experience CHE when the reason for the loss of economic activity was due to health-related factors. These results are in line with previous studies [21, 22]. If economic activity becomes impossible for health reasons, this could be directly related to increased medical costs. Therefore, it is more likely for individuals to experience CHE due to job losses at a time when medical expenses are already high. In view of these results, one can deduce that an individual’s reason for experiencing CHE is that job loss and health problems increase the chance of CHE [23]. This suggests that people who experience CHE are likely to suffer a twofold setback. In other words, as the denominator shrinks and the numerator grows, the chances of experiencing CHE increase. Additionally, according to the age subgroup analysis, the group who quit economic activities while aged 65 years or older was most likely to experience CHE [24, 25]. Therefore, there is a need to manage medical expenditures in this group because it is likely that their medical expenses will increase rapidly.

The results of the analysis of subgroups by sex and head of household suggest that for women or non-householder individuals, groups experiencing economic difficulties such as CHE try to escape their economic difficulties by changing their states of economic activity [26]. In addition, as the ratio of medical expenses in the CHE calculation increases, the OR of the inactive → active groups increases, so it is also possible that CHE may impact the group’s employment status. Therefore, further research on this phenomenon is necessary.

As such, those who have lost their ability to respond to medical expenses will be very likely to experience CHE. When health reasons, old age, and other medical costs force individuals to cease economic activities, the expense of future medical services carry a significant burden. Therefore, burdensome medical costs can lead to poverty in the long term. In addition, previous studies have demonstrated that economic conditions are a measure of an individual’s health status and quality of life. Subjective health status [27] or quality of life [25, 28] were reported to be better in better economic conditions. Economic conditions are closely related to medical access, and economic difficulties are often a matter of health equity [29]. Thus, economic hardship can lead to poor health, which, in turn, can lead to economic hardship.

Therefore, efforts should be made to prevent poverty and bankruptcy due to medical expenses. In Korea, the government enacted the Act on the Support for Catastrophic Health Expenditure in 2018 and has been implementing the Catastrophic Health Expenditure Support Program since July of the same year [30]. Support for medical expenses can help prevent poverty by eliminating the economic difficulties caused by medical expenses in case of temporary high medical expenses. However, this policy can only benefit those who meet income and property requirements. Analyses of household income levels showed that the middle class was more likely to experience CHE than the lower class. People in this blind spot will likely experience even more distressing CHE, and this is similar to the situation in the United States [2, 31].

These findings have some limitations that require attention. This study tried to consider the time perspective, but it may be difficult to ensure causality in the findings reported here. In addition, despite the efforts of investigative agencies and researchers, there may be biases such as response bias and recall bias. The respondent may have been missed if there is a serious illness among those surveyed. In addition, some items, such as current smoking status and drinking status, may have a bias because they are self-reported, and healthcare use related survey items may be skewed in data collection because they collect data retrospectively [18]. Finally, some analyses results show that the 95% confidence interval is very wide, so it is necessary to pay attention to the results interpretation. However, this study’s strength is that it analyzed the general population with representative national data. It is also meaningful that this study examined the reasons for economic inactivity and examined different criteria for CHE.

## Conclusions

In conclusion, this research shows that those who became unemployed were more likely to experience CHE than those who continued to be employed. The CHE experience was especially likely when the cause of job loss was health related. In other words, CHE was likely to be experienced when two things happened at the same time: an increase in the numerator (medical expenses) and a decrease in the denominator (disposable income). Therefore, to avoid CHE, the numerator should be reduced and the denominator should be increased. Efforts are needed to expand coverage for people suffering from high medical expenses.

## Supplementary information

**Additional file 1: Figure S1.** Sensitivity analysis of association between changes in economic activity and CHE experience by different criteria for defining CHE. All covariates were adjusted; CHE, catastrophic health expenditure; CHE experience in 2015.

## Data Availability

The dataset is available on the Korea Health Panel Survey (KHPS) website, https://www.khp.re.kr:444/eng/main.do.
