# Supplementary material for: Association between changes in economic activity and catastrophic health expenditure: findings from the Korea Health Panel Survey, 2014–2016
Source: Cost Eff Resour Alloc. 2020 Sep 16;18:36. doi: 10.1186/s12962-020-00233-9 (PMC7493960; doi:10.1186/s12962-020-00233-9)
Supplement: Supplementary file 1 — Additional file 1: Figure S1. Sensitivity analysis of association between changes in economic activity and CHE experience by different criteria for defining CHE. All covariates were adjusted; CHE, catastrophic health expenditure; CHE experience in 2015. [file 12962_2020_233_MOESM1_ESM.docx]

**
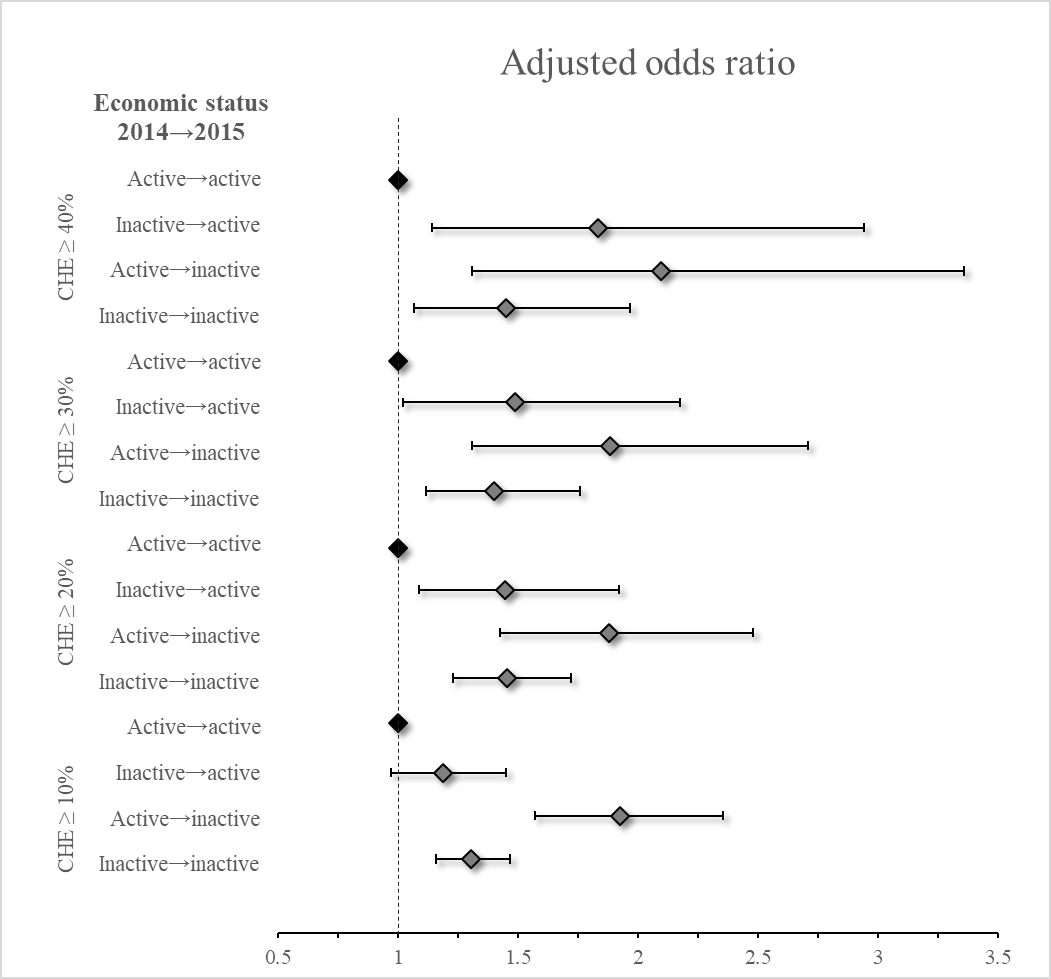
**

**Additional file 1. Figure S1:. Sensitivity analysis of association between changes in economic activity and CHE experience by different criteria for defining CHE.** All covariates were adjusted; CHE, catastrophic health expenditure; CHE experience in 2015
